# Topical Ruxolitinib and Delgocitinib Versus Systemic JAK Inhibitors: A Comparative Safety Review

**DOI:** 10.3390/ijms262110592

**Published:** 2025-10-30

**Authors:** Simone Lisa Böll, Seyed Morteza Seyed Jafari, Curdin Conrad, Christoph Schlapbach

**Affiliations:** 1 Department of Dermatology, Inselspital, Bern University Hospital, University of Bern, 3010 Bern, Switzerland; morteza.jafari@insel.ch (S.M.S.J.); christoph.schlapbach@insel.ch (C.S.); 2 Department of Dermatology and Venereology, University Hospital of Lausanne, 1005 Lausanne, Switzerland; curdin.conrad@chuv.ch; 3 Department of Dermatology and Venereology, University of Freiburg, 79085 Freiburg im Breisgau, Germany

**Keywords:** atopic dermatitis, ruxolitinib, delgocitinib, Janus kinase inhibitors, chronic inflammatory skin diseases, safety profile, systemic therapy, topical therapy

## Abstract

Janus Kinase Inhibitors (JAKi) represent a novel class of drugs for the treatment of chronic inflammatory skin diseases. Topical JAKi (t-JAKi) offer targeted therapy at potentially reduced systemic side effects and improved long-term safety compared to systemic JAKi (s-JAKi). This narrative review assesses the pharmacokinetics and safety profile of currently available t-JAKi, ruxolitinib and delgocitinib, with a comparative analysis to s-JAKi. Pharmacokinetic data show that topical ruxolitinib achieves effective dermal concentrations, with minimal systemic exposure. Clinical trials consistently report low rates of adverse events, primarily application-site reactions, while systemic events such as upper respiratory tract infections occur at rates comparable to placebo. Data on delgocitinib similarly indicate negligible systemic absorption and a favorable safety profile. Taken together, these findings suggest that t-JAKi may represent safer alternatives to s-JAKi for selected patients with localized inflammatory skin diseases, particularly those with comorbidities or heightened systemic risk. Long-term studies and real-world evidence are needed to confirm sustained safety and guide their optimal integration into clinical practice.

## 1. Introduction

Janus Kinase Inhibitors (JAKi) represent a novel class of therapeutics for the treatment of numerous inflammatory, autoimmune and myeloproliferative disorders, demonstrating remarkable efficacy within the last years. They are a family of targeted synthetic drugs that elicit their therapeutic effects in vivo by inhibiting Janus kinases (JAK), a family of four intracellular tyrosine kinases—JAK1, JAK2, JAK3, and TYK2—which mediate signaling downstream of a broad range of cytokine receptors. Upon cytokine binding, JAKs associated with the receptors become activated, leading to the phosphorylation of the signal transducer and activator of transcription (STAT) proteins, which subsequently translocate to the nucleus to regulate the transcription of inflammatory genes and orchestrate immune responses.

JAK-STAT signaling is essential for cytokines of the type I and II families. Thus, JAKi inhibit a broad range of cytokines and have shown clinical efficacy in inflammatory diseases mediated by Th1, Th2, and Th17, which are central to the pathogenesis of many inflammatory conditions. Furthermore, their demonstrated clinical efficacy extends beyond classic inflammatory responses to include roles in certain granulomatous and fibrotic diseases, highlighting the influence of JAK-STAT pathways in diverse pathological processes. Advances in our understanding of these cellular and molecular pathways, along with the identification of key cytokines that drive the pathology of inflammatory, autoimmune and myeloproliferative diseases, have unequivocally paved the way for the application of JAKi in these conditions.

JAKi differ in selectivity and mode of administration. They are available as selective, partially selective and unselective inhibitors (pan-JAKi). This spectrum of selectivity may influence their therapeutic efficacy and safety, as different JAK isoforms are predominantly associated with signaling from different cytokine receptors. In terms of administration, JAKi can be delivered systemically, in oral formulations (s-JAKi), or through topical formulations (t-JAKi). T-JAKi penetrate the epidermis and inhibit JAKs in keratinocytes and resident immune cells, leading to reduced cytokine signaling and decreased local inflammation. Crucially, this mechanism operates via the same intracellular pathway as s-JAKi [1,2,3,4,5,6].

While the clinical efficacy of s-JAKi in chronic inflammatory skin diseases is well established, potentially severe adverse events—particularly cardiovascular, thromboembolic, and oncologic risks—remain a matter of concern. These limitations have driven growing interest in t-JAKi as a promising alternative that may reduce systemic risks while maintaining local efficacy by focusing on pharmacologic action within the skin.

This narrative review explores the pharmacokinetic and safety distinctions between t-JAKi and their systemic counterparts, with the aim of clarifying their respective roles. It analyzes available literature and clinical trial data to provide a comprehensive understanding of the pharmacokinetics, distinct safety profiles, and adverse events of t-JAKi compared to their systemic counterparts. We aim to elucidate the nuanced risk-benefit assessment for t-JAKi and s-JAKi, thereby informing clinical decision-making and highlighting critical areas for future research in the evolving landscape of JAKi therapy for chronic inflammatory skin diseases.

A comprehensive literature review was conducted to identify relevant publications on t-JAKi and s-JAKi, with a primary focus on pharmacokinetic and safety data in the treatment of inflammatory skin diseases. The PubMed^®^ database and ClinicalTrials.gov were searched from January 2020 to May 2025. Search terms included “systemic JAK inhibitor”, topical JAK inhibitor”, “ruxolitinib”, “delgocitinib”, “JAK inhibitor safety”, “pharmacokinetics JAK inhibitors” and “adverse events JAK”. Peer-reviewed original articles, including preclinical and Phase 1–3 clinical study results, were included. Studies without safety data or focusing on non-dermatological indications, case reports, reviews, commentaries, and articles with no English text were excluded. The analysis is based on prior studies, and did not involve any new investigations on human subjects or animals.

## 2. Clinical Landscape of Topical and Systemic JAK Inhibitors

In recent years, several s-JAKi have been approved for the treatment of atopic dermatitis (AD), plaque psoriasis (PP), and alopecia areata (AA) (Table 1). These approvals mark a paradigm shift in dermatological therapy, introducing effective oral options for diseases historically managed with conventional immunosuppressants or biologics. Beyond approved indications, s-JAKi are also demonstrating considerable efficacy in a growing number of other cytokine-mediated dermatological diseases. [1,2,4,7,8,9,10].

Common treatment-emergent adverse events observed with s-JAKi include upper respiratory tract infections, nasopharyngitis, and herpes virus reactivation. Other frequent adverse events comprise headache and acne (Table 2). Hematologic and metabolic laboratory abnormalities are also observed, such as decreases in hemoglobin, absolute lymphocyte and neutrophil counts, as well as elevated lipids and creatine phosphokinase [7,15,16,17,18].

Among s-JAKi, tofacitinib, a preferential JAK1/3 inhibitor, has been used in rheumatoid arthritis (RA) since 2012, providing the most extensive long-term safety dataset to date. Concerns about systemic safety escalated in 2021, when the U.S. FDA issued a black box warning following results of the post-marketing ORAL Surveillance trial in RA patients [19,20]. The ORAL Surveillance study demonstrated increased risks of major cardiovascular events (MACE), venous thromboembolisms (VTE; including deep vein thrombosis and pulmonary embolism), and malignancy (excluding non-melanoma skin cancer [NMSC]) in RA patients aged 50 years or older with at least one cardiovascular risk factor, compared to patients receiving tumor necrosis factor inhibitor (TNFi) therapy. The warning, initially specific to tofacitinib, was later expanded to non-pan-JAKi such as baricitinib and upadacitinib, despite limited confirmatory data [4,18,19].

Caution must be exercised when extrapolating these findings from RA patients to dermatologic populations. Patients with AD or AA are typically younger and have fewer baseline cardiovascular risk factors than the RA cohort examined in the ORAL surveillance study. Furthermore, the risk profiles of individual JAKi differ depending on their selectivity for JAK1, JAK2, and JAK3.

In contrast, available evidence on upadacitinib in AD, based on comprehensive studies with follow-up periods extending up to 2.75 years (median 1.62 years), has so far shown no increased risk for VTE (Table 2). Nevertheless, uncertainties persist regarding the potential increased risk for malignancy (including NMSC), MACE, and serious infections [15]. Overall, the long-term safety of s-JAKi in dermatologic use remains to be fully defined.

Despite encouraging short-term efficacy and tolerability, cumulative clinical experience with s-JAKi in dermatologic indications—particularly in younger patient populations—remains limited. This lack of long-term data underscores the need for cautious prescribing, regular monitoring, and ongoing real-world pharmacovigilance [1,2].

**Table 2:** Common adverse events in s-JAKi and t-JAKi (selected pivotal studies).

Drug	Indication	*n*	Any AE (%)	Serious AE (%)	Naso- pharyn- gitis (%)	Herpes zoster (%)	NMSC (%)	Acne (%)	Application Site Reaction
**s-JAKi**									
Abrocitinib 100/200 mg	AD [21]	261	51–62	0.9–2.5	6.6–9.2	0.8–1.8	NR	2.9–6.6	N/A
*Placebo*		131	53	3.8	6.9	0	NR	0	N/A
Baricitinib 1/2 mg	AD [22]	292	51–54	0.7–1.4	2–4.8	0–0.7		NR	N/A
*Placebo*		146	49	2	7.5	0		NR	N/A
**t-JAKi**									
Ruxolitinib 0.75–1.5%	AD infants [23]	277	34–67	<2	2.6–3	NR	NR	NR	14–15
*vehicle*		83	33	0.8	0.8	NR	NR	NR	2.8–4.8
Delgocitinib 0.25–0.5%	AD in infants [13]	22	95.5	4.5	72.7	NR	NR	NR	0
Delgocitinib 20 mg/g		639	45–46	2	7	NR	NR	NR	NR
*vehicle*	CHE [12]	162	45–51	2	6–9	NR	NR	NR	NR

*n* = number of participants, N/A = not applicable, NR = not reported.

In contrast, t-JAKi have recently emerged as a promising alternative. Ruxolitinib has been approved for AD and vitiligo, and delgocitinib for AD (Japan) and chronic hand eczema (CHE) [14,17,24] (Table 1). Ongoing trials are investigating the efficacy of t-JAKi in PP, AA, and chronic graft-versus-host-disease (cGVHD) [25]. Given the potentially serious systemic adverse events associated with s-JAKi and the persistent uncertainties regarding their long-term safety, t-JAKi offer a compelling therapeutic strategy—potentially maintaining efficacy while markedly reducing systemic exposure.

## 3. Topical Ruxolitinib and Delgocitinib Versus Systemic JAK Inhibitors

### 3.1. Ruxolitinib

Ruxolitinib is the only compound currently approved for both systemic and topical administration. As a JAK1/2 inhibitor, it modulates signaling pathways crucial for various inflammatory and myeloproliferative processes. Its systemic formulation has well-established indications in hematological malignancies and immune dysregulation, targeting conditions such as myelofibrosis, polycythaemia vera, and chronic or acute graft-versus-host-disease (GvHD). In these contexts, its inhibition of JAK1 and JAK2 pathways is critical for controlling abnormal cell proliferation and immune responses.

More recently, ruxolitinib has emerged as a significant therapeutic option in dermatology, with its topical formulation offering a targeted approach to inflammatory skin diseases. It is currently approved by the FDA for the treatment of mild-to-moderate AD, including children aged 2 years and older since September 2025, and non-segmental vitiligo in adults and adolescents aged 12 and older. This section will detail the pharmacokinetic and safety profile of topical ruxolitinib in dermatological conditions. [11,14,17,24,25,26,27,28,29,30].

#### 3.1.1. Pharmacokinetics of Topical Ruxolitinib

Pharmacokinetic investigations demonstrate a stark difference in systemic exposure between t-JAKI and s-JAKI. In a preclinical study with mini pigs, the oral administration of ruxolitinib resulted in significantly higher (approx. 38-fold greater) plasma concentrations compared to topical application (Table 3) [28]. Conversely, the same investigation revealed that intradermal concentrations were 507-fold higher with topical application compared to oral administration. This indicates the ability of topical ruxolitinib to reach therapeutic levels precisely within the skin, while profoundly limiting systemic absorption into the bloodstream. The study further elucidated a critical point: dermal concentrations achieved from oral administration may be insufficient to effectively inhibit local JAK-STAT signaling pathways within the skin, potentially resulting in suboptimal therapeutic efficacy in dermatological conditions compared to direct topical application.

These compelling preclinical findings are strongly corroborated by robust data from clinical phase I-III studies of topical ruxolitinib in patients with AD and vitiligo. These large-scale trials, which primarily involved application to a maximum of 20% body surface area (BSA), consistently demonstrated minimal systemic exposure. For instance, the total systemic exposure over 12 h (AUC_0–12_) for topical 1.5% ruxolitinib cream applied to ≤20% BSA was quantified as 683 hnM. This systemic exposure level amounted to approximately only 25% of the total systemic drug exposure (2716 hnM) observed following a standard 15 mg twice daily oral administration in healthy participants [24]. Importantly, plasma concentrations did not increase proportionally with cream strength, suggesting a saturation of systemic absorption at therapeutic concentrations and a favorable absorption ceiling.

Further covariate analysis identified the dose of cream applied, percentage of BSA treated, and the baseline Investigator’s Global Assessment (IGA) score as the most influential factors affecting steady-state concentration (C_ss_). Crucially, despite these minor variations in systemic exposure, there was no significant correlation between plasma concentrations and hematological changes commonly associated with sJAKi, with the exception of a transient platelet increase in some patients. Overall, the consistently low plasma concentrations achieved with topical ruxolitinib are not expected to reach levels associated with the systemic adverse events commonly observed with s-JAKi, thereby strongly supporting its favorable local and systemic safety profile [14,24,26,29].

**Table 3:** Overview of pharmacokinetic parameters after systemic and topical administration of ruxolitinib in minipigs [28].

	Systemic Ruxolitinib	Topical Ruxolitinib
dose	40 mg/kg bodyweight	1.5% cream applied to 10% BSA
Cmax (nM)	153 ± 173	3.98 ± 3.5
AUC (h*nM)	1060 ± 1050	34.6 ± 23.4
Css (nM)	n/a	2.7 ± 1.8 nM
Tmax (h)	3.3 ± 1.5	3.5 ± 3.3

#### 3.1.2. Adverse Events of Topical Ruxolitibib

The safety profile of topical ruxolitinib has been comprehensively assessed across Phase 3 clinical trial programs for atopic dermatitis (TRuE-AD1/2) and non-segmental vitiligo (TRuE-V1/2), as well as their long-term extension studies, providing robust data on its tolerability. The overall findings consistently indicate that topical ruxolitinib is well-tolerated, with a distinct safety profile compared to systemic JAK inhibitors, primarily due to its lower systemic absorption.

Across these studies, various cutaneous reactions and adverse events were reported in patients receiving topical ruxolitinib for AD. A significant proportion (range 34–67%) experienced treatment-emergent adverse events. Importantly, the vast majority of these were mild to moderate in severity and their frequency was generally comparable to, or not significantly higher than, those observed with vehicle (placebo) application. The most common cutaneous reactions were presented in Ref. [29].

The most commonly reported adverse events included upper respiratory infections, nasopharyngitis and headaches. These events were typically mild and self-limiting, and their incidence rates were consistent with those expected in the general population or in similar patient cohorts receiving the vehicle. Importantly, serious infections were rare throughout the studies, and were not deemed to be causally linked to ruxolitinib treatment, a crucial distinction from s-JAKi.

The most frequent cutaneous reactions included application-site pain or pruritus and application-site acne (approximately 5% in [29]). These local reactions were typically mild to moderate and generally resolved with continued treatment or did not necessitate discontinuation. Localized infections, such as those caused by herpes simplex virus (HSV) and molluscum contagiosum, were also reported [14,24,29]. While these viral infections can occur in dermatological patients, their incidence rates were generally low and managed effectively, without raising concerns of widespread immunosuppression at the local level.

Long-term safety assessments, derived from open-label extension phases of the two Phase 3 studies that followed patients for up to 52 weeks, have further reinforced the safety profile. For instance, in pooled analyses of the AD trials, MACE were reported in only two patients out of a large cohort with extended follow-up, both of whom had pre-existing cardiovascular risk factors, and these events were deemed unrelated to ruxolitinib treatment. Similarly, a VTE was reported in only one patient, also considered unrelated to the study drug. Furthermore, NMSC were identified in five patients during the long-term follow-up; critically, four of these five NMSC cases occurred outside the application site, and all were considered unrelated to the treatment [14].

### 3.2. Delgocitinib

Delgocitinib is a pan-JAKi that was approved in Japan in 2020 for the treatment of atopic dermatitis (AD), making it the first t-JAKi available. Unlike ruxolitinib, delgocitinib is exclusively available as a topical formulation. While earlier reports indicated an oral formulation was under clinical development in Japan for systemic autoimmune disorders, the current clinical focus and regulatory approvals for delgocitinib remain squarely on its topical delivery.

In 2024, it was further approved by the European Medicines Agency (EMA) for the treatment of moderate to severe CHE. Beyond AD, delgocitinib has been investigated in AA, with ongoing trials exploring further indications [13,31,32,33,34,35,36].

#### 3.2.1. Pharmacokinetics of Topical Delgocitinib

Published data on the pharmacokinetic profile of topical delgocitinib is scarce. No data exist on its use across maximum BSA. A phase 3 study involving Japanese infants with AD aged 6 to <24 months treated with 0.25% or 0.5% delgocitinib ointment, applied to 5–30% BSA twice daily for up to 52 weeks, showed low systemic exposure [13]. Specifically, delgocitinib was not detected in the plasma of most infants (68.2–95.2%) across various time points and doses. Importantly, the study confirmed no evidence of drug accumulation with prolonged use. Infants, due to their higher surface area-to-volume ratio, thinner stratum corneum, and developing skin barrier function, are generally more susceptible to systemic absorption of topical medications than older children or adults [37]. The very low systemic exposure in this sensitive cohort strongly indicates that delgocitinib ointment is unlikely to increase the risk of systemic adverse events, irrespective of age or the duration of treatment within the studied parameters.

Further reinforcing these observations, a recent study investigated systemic exposure and relative bioavailability of delgocitinib cream in patients with moderate to severe CHE. This open-label, single-arm Phase 1 study involved adult patients applying 20 mg/g (2%) delgocitinib cream twice daily for one week, with pharmacokinetic sampling on Day 1 and Day 8. The results were compared with data from two Phase 1 trials involving oral delgocitinib in healthy adults (1.5–12 mg and 1–100 mg). The study demonstrated no to minimal systemic exposure of delgocitinib cream, with no significant accumulation of delgocitinib observed over the study period. Systemic exposure with topical application showed to be significantly lower than with oral administration. The relative bioavailability of topical delgocitinib cream compared to oral administration was remarkably low, approximately 0.6% [35].

Critically, plasma concentrations achieved with topical application were consistently over 40-fold below the drug’s in vitro half-maximal inhibitory concentration (IC_50_) in human whole blood. For the lowest, subtherapeutic dose of delgocitinib tested (1.5 mg), systemic exposure levels were more than 10-fold lower with topical delgocitinib cream than oral delgocitinib. These findings suggest that topical use is highly unlikely to exert a meaningful systemic JAK inhibitory effect, thereby mitigating concerns for class-specific systemic adverse events typically associated with oral JAKi.

#### 3.2.2. Adverse Events of Topical Delgocitinib

The adverse event profile of topical delgocitinib, as observed across various clinical studies for AD, CHE, and AA, consistently reflects its localized action and minimal systemic exposure. Overall, topical delgocitinib demonstrates favorable tolerability, with the vast majority of reported adverse events being mild to moderate in severity.

In the adult studies evaluating its efficacy in CHE (e.g., DELTA 1 and DELTA 2 trials) and AD, treatment-emergent adverse events were reported in a range of 45–68.3% of patients. Notably, the incidence rates of these events were often comparable to those observed with placebo or vehicle cream, indicating that many were background events unrelated to the active treatment [32,33,34]. Similarly to ruxolitinib, the most common adverse events included nasopharyngitis and headaches, consistent with common occurrences in the general population. Notably, worsening of chronic hand eczema was reported in approximately 5% of patients in some CHE trials [33]. While this could represent local irritation, it was generally mild and transient, with overall tolerability improving over time (e.g., stinging/burning sensations decreasing from 83.9% at week 1 to 90.2% at week 36 in the DELTA 3 extension study for CHE). All treatment-related adverse events were mild and did not lead to significant discontinuations, further underscoring the cream’s manageability [32,33,34].

The Japanese study involving infants with AD, despite the absence of a placebo control group for direct comparison, also revealed a favorable safety profile. Adverse events occurred in 21 of 22 infants (95.5%), a high percentage primarily reflecting the comprehensive capture of any health event in this vulnerable and usually otherwise healthy age group. The majority of these events were mild, with nasopharyngitis (72.7%) and impetigo (27.3%) being the most common, both frequently observed in infants and young children. Importantly, no treatment-related adverse events were reported. The incidence of adverse events did not increase over time, suggesting no dose-dependent or cumulative increase in risk with prolonged use [13].

In the long-term safety assessments of delgocitinib skin atrophy or telangiectasia, common and problematic adverse effects associated with the long-term use of topical corticosteroids were visually not observed. This distinction positions topical delgocitinib as a highly attractive non-steroidal alternative for chronic dermatoses requiring sustained therapy, particularly on sensitive skin areas like the face and intertriginous zones. Furthermore, unlike some calcineurin inhibitors, delgocitinib has not been associated with significant localized irritation, such as burning or stinging sensations, beyond the initial mild and transient reports [13].

Regarding systemic safety, the adult CHE study reported no adverse events. Delgocitibib cream was well tolerated, and no adverse events were reported during the trial period. More broadly, in the pivotal DELTA 1, DELTA 2, and DELTA 3 (long-term extension) trials for CHE involving hundreds of patients, no new safety concerns emerged. Crucially, there were no reports of hematologic adverse events, MACE, VTE, or malignancies (including NMSC) that were deemed related to treatment [12,38]. The consistent finding of negligible systemic drug exposure aligns with this excellent systemic safety profile, strongly suggesting that topical delgocitinib does not carry the same systemic risks that are class warnings for oral JAK inhibitors [35].

## 4. Conclusions and Future Direction

Based on the two reviewed t-JAKi, t-JAKi represent a significant advance in the management of chronic inflammatory skin diseases, primarily by preserving robust therapeutic efficacy while profoundly reducing the incidence of systemic adverse events commonly associated with their oral counterparts. This enhanced safety margin significantly expands their clinical utility, particularly for patients who may have limited tolerance for systemic immunomodulation or who require long-term treatment.

The distinct pharmacokinetic profiles of t-JAKi are central to this improved safety. Topical ruxolitinib achieves effective dermal concentrations while reducing systemic exposure, resulting in systemic drug levels substantially lower than those observed with therapeutic doses of oral ruxolitinib [24]. Similarly, delgocitinib, currently available solely as a t-JAKi, demonstrates negligible systemic absorption. This markedly reduced systemic exposure results in a broad safety margin for systemic adverse events.

Consequently, the adverse events reported with t-JAKi were low in incidence and predominantly confined to mild-to-moderate application-site reactions, with no relevant systemic laboratory changes observed (Table 2). Importantly, the serious systemic adverse events that are class warnings for s-JAKi–such as serious infections, MACE, or VTE—were either not observed or deemed unrelated to treatment in studies of topical ruxolitinib and delgocitinib. This combination of targeted local action and minimal systemic drug levels positions topical JAK inhibitors as a particularly suitable treatment option for patients at risk of cardiovascular disease, neoplasia, or other systemic comorbidities, thereby significantly broadening the applicability of the JAK inhibitor class in dermatology.

Regarding the concern of NMSC risk with s-JAKi, long-term assessment with t-JAKi use is crucial. Only a few isolated cases of NMSC (e.g., one application-site NMSC for ruxolitinib [14]) have been observed across all topical studies to date, and direct causality to t-JAKi has not been established. Most occurred in patients with pre-existing risk factors like prior UV exposure or NMSC history. Future studies with extended follow-up are necessary to fully evaluate this potential long-term risk.

Both s-JAKi and t-JAKi play important, complementary roles in clinical practice, depending on disease extent and severity. Efficacy data (e.g., Investigator Global Assessment [IGA] and Eczema Area and Severity Index [EASI] scores in AD) suggest that t-JAKi can achieve outcomes comparable to systemic therapies in localized disease. However, for patients with more extensive involvement, the practicality of applying topical treatments becomes a limiting factor, and s-JAKi may be more appropriate to ensure compliance and adequate coverage. Furthermore, in diseases with systemic inflammatory activity or associated comorbidities (e.g., psoriasis arthritis), driven by complex cytokine cascades, t-JAKi may not provide sufficient systemic inflammatory control, making s-JAKi the more appropriate therapeutic choice. 

Comparing t-JAKi and s-JAKi, as well as different t-JAKi, poses inherent challenges. Direct comparative clinical data are limited. Variations in patient populations, study designs, observation periods, and evolving clinical experience further hinder definitive conclusions. Importantly, a notable indication bias exists: t-JAKis are primarily utilized for localized disease, whereas s-JAKi are reserved for severe or refractory cases that inherently represent a higher-risk cohort. This difference complicates direct comparisons of systemic safety.

The use of t-JAKi is generally limited to ≤20% body surface area (BSA). Application to eroded skin, sensitive regions, or larger BSA may increase systemic absorption—an important consideration when extrapolating trial safety data to real-world practice. Moreover, in everyday use, factors such as sweating, friction from clothing, or patient movement may reduce the residence time of topical formulations on the skin, potentially lowering effective exposure compared to controlled clinical settings.

Furthermore, our review relies on published data, which may be subject to reporting biases, and comprehensive long-term safety data for t-JAKi is still emerging. Therefore, exploring combined therapy strategies—such as systemic induction followed by targeted topical maintenance—is a critical future direction to optimize efficacy while mitigating prolonged systemic exposure.

In conclusion, this comparative safety review highlights that the t-JAKi ruxolitinib and delgocitinib represent a promising new treatment modality for chronic inflammatory skin diseases. Their favorable systemic safety profile, driven by localized pharmacologic action and minimal systemic absorption, positions them as attractive alternatives to s-JAKi, in particular for patients with localized disease or cardiovascular, hematologic, or oncologic risk factors. As regulatory approvals and indications for t-JAKi continue to increase globally, further clinical and real-world data will be critical to clarify the long-term safety of t-JAKi. In this context, t-JAKi represent an important advancement in the management of inflammatory skin diseases, particularly for patients in whom systemic risks must be minimized.

## Figures and Tables

**Table 1:** Currently approved JAK inhibitors for dermatological indications [11,12,13,14].

Drug	Mode of Administration	Selectivity	Approved Indications	Region of Approval	Year of First Approval
**s-JAKi**					
Abrocitinib	oral	JAK1	AD ^1^	USA, EU, Japan	2021 (EU/Japan)
Baricitinib	oral	JAK1/2	AD, AA	USA, EU	2017 (EU/Japan)
Deucravacitinib	oral	TYK2	PP	USA, EU	2022 (USA)
Deuruxolitinib	oral	JAK2/1	AA	USA	2024 (USA)
Ritlecitinib	oral	JAK3	AA	USA	2023 (USA)
Upadacitinib	oral	JAK1	AD	USA, EU, Japan	2019 (all)
**t-JAKI**					
Ruxolitinib cream	topical	JAK1/2	AD, Vitiligo	USA, EU	2021 (USA)
Delgocitinib ointment	topical	Pan-JAK	AD CHE	Japan EU	2020 (Japan) 2024

^1^ in adults and children ≥ 12 years.

## Data Availability

No new data were created or analyzed in this study.

## Abbreviations

The following abbreviations are used in this manuscript:

JAKi	Janus Kinase Inhibitors
s-JAKi	systemic Janus Kinase Inhibitor
t-JAKi	topical Janus Kinase Inhibitor
STAT	signal transducer and activator of transcription
AA	Alopecia Areata
AD	Atopic Dermatitis
AE	Adverse Event
PP	Plaque Psoriasis
FDA	Food and Drug Administration
TYK	Tyrosine Kinase
MACE	major cardiovascular events
VTE	venous thromboembolisms
RA	rheumatoid arthritis
NMSC	nonmelanoma skin cancers
CHE	chronic hand eczema
GVHD	graft-versus-host-disease
cGVHD	chronic graft-versus-host-disease
BSA	body surface area
C_ss_	steady-state concentration
IGA	Investigator’s Global Assessment
MF	Myelofibrosis
EASI	Eczema Area and Severity Index
C_max_	maximum plasma concentration
AUC	area under the curve
EMA	European Medicines Agency
T_max_	time to maximum concentration
n/a	not applicable
CPK	creatine phosphokinase
